# RacGAP1 promotes the malignant progression of cervical cancer by regulating AP-1 via miR-192 and p-JNK

**DOI:** 10.1038/s41419-022-05036-9

**Published:** 2022-07-12

**Authors:** Tianli Zhang, Chunyan Wang, Kun Wang, Ying Liang, Ting Liu, Liping Feng, Xingsheng Yang

**Affiliations:** 1 Department of Obstetrics and Gynecology, Qilu Hospital of Shandong University, Jinan, Shandong 250012 People’s Republic of China; 2 Key Laboratory of Gynecologic Oncology of Shandong Province, Qilu Hospital of Shandong University, Jinan, Shandong 250012 People’s Republic of China

**Keywords:** Cervical cancer, Cervical cancer

## Abstract

Cervical cancer (CC) is the most frequently diagnosed genital tract cancer in females worldwide. Rac GTPase-activating protein 1 (RacGAP1) is one of the specific GTPase-activating proteins. As a novel tumor protooncogene, overexpression of RacGAP1 was related to the occurrence of various tumors, but its function in CC is still unclear. In this study, bioinformatics analyses showed that RacGAP1 might be a key candidate gene in the progression of CC. RacGAP1 was significantly overexpressed in CC tissues. High RacGAP1 expression was positively associated with poor prognosis. Downregulating RacGAP1 significantly inhibited the proliferation, migration, and invasion of CC cells, while overexpressing RacGAP1 had the opposite effects. Further research showed that miR-192, which plays as a tumor suppressor in CC, was identified as a downstream target of RacGAP1 in CC cells. miR-192 inhibition could partially rescue the decrease in cell proliferation, migration, and invasion caused by RacGAP1 downregulation. In opposite, miR-192 overexpression could decrease the promotion of malignant progression caused by RacGAP1 upregulation. Mechanism studies revealed that RacGAP1 could regulate the expression and phosphorylation of c-Jun, which was the component of AP-1, via miR-192 and p-JNK separately. These findings suggested that RacGAP1 promoted tumorigenicity, migration, and invasion of CC. Therefore, it represented a potential novel prognostic marker in CC and may probably be a therapeutic target.

## Introduction

As reported in GLOBOCAN 2020, cervical cancer (CC) was the fourth most frequently diagnosed cancer and the fourth leading cause of cancer death in women worldwide. In 2020, there were an estimated 604,000 new cases and 342,000 deaths worldwide annually [1]. Although China had carried out CC screening programs and human papillomavirus (HPV) vaccinations, CC remained the most common cause of cancer-related death among Chinese female reproductive system tumors [2]. HPV is a necessary but not sufficient cause of CC [3], and the exact molecular mechanisms between HPV infection and the transition from intraepithelial lesion to invasive carcinoma were still unclear [4]. Tumorigenesis arises as a consequence of a breakdown in the balance between protooncogene and tumor suppressor genes, which leads to changes in cell proliferation and abnormal interactions between cells and their surroundings [5]. Besides, more research is needed to better predict the prognosis of CC.

Rac GTPase-activating protein 1 (RacGAP1), one of the specific GTPase-activating proteins (RhoGAP), which stimulate the intrinsic GTPase activity of Rho proteins and restore them to the inactive state of binding to GDP [6], can regulate Rac1 and CDC42 proteins to drive tumor growth [7]. RacGAP1 is essential for cytokinesis [8], and knockout of RacGAP1 results in the formation of multinucleated cells and failure of cytokinesis [9, 10]. Several authors reported that RacGAP1 is overexpressed in many different types of tumor tissues and associated with poor prognosis [11–15]. What's more, RacGAP1 was also involved in cell proliferation, transformation, motility, migration, and metastasis [16–18]. It may also be a new target in developing novel chemotherapy drugs [19]. However, the expression and definite functions of RacGAP1 in CC have not been investigated.

Here, we hypothesized that RacGAP1 was involved in the development of CC and may therefore be a potential prognostic predictor for CC patients. RacGAP1 was remarkably overexpressed in CC tissues and was associated with a poor prognosis of CC. Through in vitro and in vivo experiments, we demonstrated that RacGAP1 promoted the malignant progression of CC, and these effects on biological behavior were achieved by regulating AP-1 via miR-192 and p-JNK. Our results demonstrated a novel correlation among tumor suppressor p53 and miR-192, MKK4/7/JNK/AP-1 axis, and RacGAP1 in CC and suggested that RacGAP1 played an important role in the progression of CC and might become a new therapeutic target for CC.

## Materials and methods

### Human CC specimens

The collection of 131 paraffin-embedded primary CC samples staging from Ia1 to IIIb (FIGO stage, 2018) was authorized by the Department of Pathology, Qilu Hospital of Shandong University, between January 2008 and December 2012. Moreover, 64 primary CC tissues and matched adjacent nontumor specimens were obtained immediately after surgical resection from patients in the Department of Obstetrics and Gynecology, Qilu Hospital of Shandong University, from January 2017 to December 2018. This study was examined and approved by the Medical Ethics Committee of Qilu Hospital of Shandong University (KYLL-2017-539). The specimens were used with informed consent from the patients.

### Statistical analyses

The quantitative data with normal distribution were presented as mean ± SEMs. Comparison between groups was estimated by Students’ *t* test or one-way analysis of variance, as was appropriate. Each experiment was repeated three times. The Pearson *χ*^2^ test was used to evaluate the relationship between RacGAP1 expression and clinicopathological parameters in 131 patients with CC. The two-tailed Spearman correlation analysis was applied to explore the correlation between RacGAP1 expression and clinicopathological features. The survival analysis was conducted by the Kaplan–Meier method with a log-rank test. Cox’s proportional hazard regression model was used to analyze the risk factors associated with the prognoses of these patients. Wilcoxon matched-pairs signed-rank test was performed to compare RacGAP1 mRNA and protein expressions between CC tissues and adjacent normal tissues. Differences were considered statistically significant when *p* < 0.05 (*p < 0.05, **p < 0.01, ***p < 0.005, and ****p < 0.001). All the analyses above were performed using IBM SPSS Statistics 24.0 (Armonk, NY).

### Supplemental methods

Bioinformatics analyses were performed in R language. mRNA expression was measured using qRT-PCR. Protein expression was detected by Western Blot. Immunohistochemistry staining was used to test the protein expression in paraffin tissue sections. Immunofluorescence staining was used to detect RacGAP1 location in CC cells. Cell proliferation was evaluated using the CCK8, EdU incorporation, and clone formation assay. Transwell assay and wound healing assay were used to assess cell migration and invasion. Tumor xenograft models were established to test in vivo tumorigenesis. RNA immunoprecipitation (RIP) was used to analyze RNA molecules binding with RacGAP1. To detect active RhoA, immunoprecipitation of active RhoA was performed with the RhoA Activation Assay Kit. Details of the materials and methods were shown in Supplementary Materials 1. Primer sequences are listed in supplementary materials 2.

## Result

### RacGAP1 might be a key candidate gene in the progression of cervical cancer

**Fig. 1:**
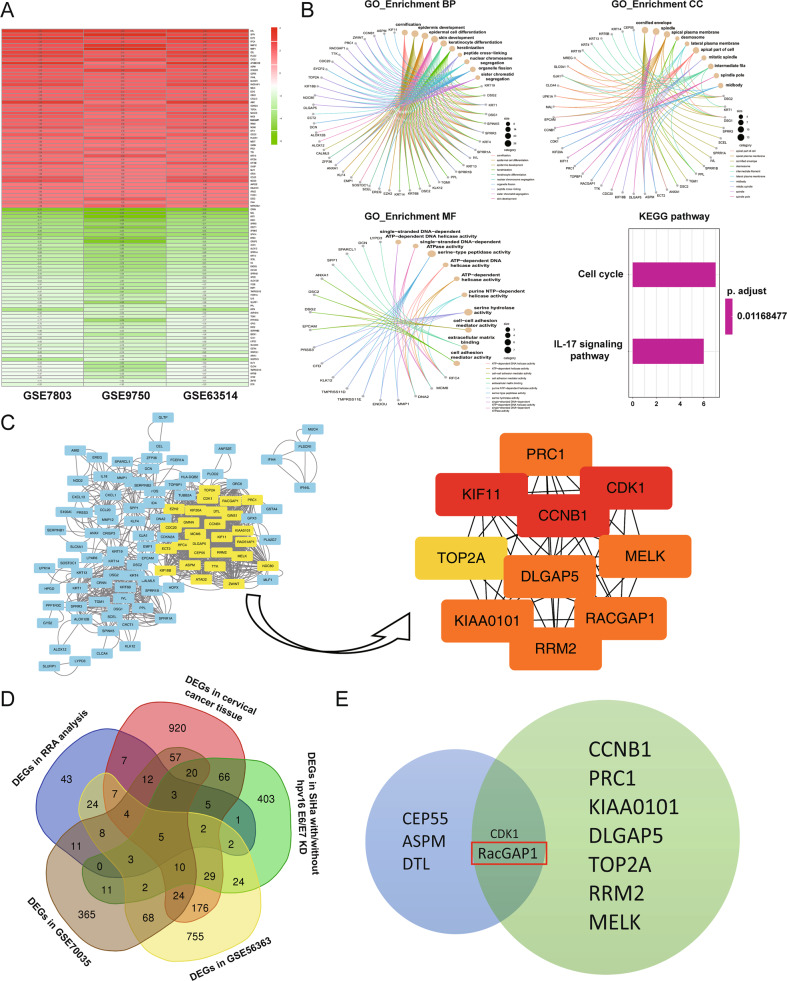
Bioinformatics analyses of cervical cancer. **A** Identification of robust DEGs of GSE7803, GSE9750, and GSE63514 by RRA analysis. Heatmap showing the top 50 upregulated genes and top 50 downregulated genes according to logFC. Each row represented one gene and each column indicates one dataset. Red indicates upregulation and green represents downregulation. The numbers in the heatmap indicate logarithmic fold change in each data set calculated by the “limma” R package. **B** GO analysis and KEGG analysis for DEGs from RRA analysis. **C** PPI network and the hub gene module of DEGs. **D** DEGs among the mRNA expression profiling datasets RRA analysis, cervical cancer tissue, SiHa with/without HPV16 E6/E7 KD, GSE56363, and GSE70035. An overlap of five genes was observed among the five data sets. **E** Two genes were found by overlapping 10 PPI hub-genes and five hub DEGs. DEG differentially expressed gene, GEO Gene Expression Omnibus, RRA robust rank aggregation, GO Gene Ontology, KEGG Kyoto Encyclopedia of Genes and Genomes. PPI protein–protein interaction, CDK1 cyclin-dependent kinase 1, RacGAP1 Rac GTPase-activating protein 1.

GSE7803, GSE9750, and GSE63514 three datasets showed the gene expression profiles on paracancerous normal samples and tumor samples of CC. Following the standardization of the microarray results, we acquired 684 differentially expressed genes (DEGs) in GSE7803 dataset, 771 DEGs in GSE9750 dataset, and 684 DEGs in GSE63514 dataset. Based on the result of the RRA analysis, a total of 61 upregulated and 76 downregulated significant DEGs were identified between normal and cancerous tissues. Fig. 1A heatmap showed the top 50 upregulated and downregulated DEGs.

To determine the biological significances of the 137 DEGs, functional and pathway enrichment analyses were performed using the R package “clusterprofiler”. Results of GO analysis and KEGG pathway enrichment analysis were shown in Fig. 1B.

To analyze the interactions among the DEGs and find hub-genes, PPIs were constructed using Cytoscape software. According to the PPI networks, the top ten genes with highest interaction degrees were KIF11, CCNB1, CDK1, PRC1, DLGAP5, MELK, KIAA0101, RacGAP1, RRM2, and TOP2A (Fig. 1C).

140 DEGs were identified from HPV16-positive CC tissues and HPV16-negative normal tissues, as well as SiHa cells with or without HPV16 E6/E7 knockdown in two data sets [20]. Besides, GSE56363 focused on the gene expression between CC samples with a 6-month complete response (12 patients) and non-complete response (nine patients). GSE70035 showed gene expression profiles on six samples of neoadjuvant chemotherapy responders and six samples of non-responder in CC. Five genes were overlapped among the DEGs from the four data sets and the RRA analysis (Fig. 1D). Finally, we identified two genes by overlapping the five genes obtained before and 10 hub-genes from PPI networks. They are CDK1 and RacGAP1 (Fig. 1E). They may be the key genes in the progression of CC. The biological functions of RacGAP1 in CC are unknown. So, we intended to study whether the abnormal expression of RacGAP1 was related to cervical carcinogenesis.

### Expression of RacGAP1 in human cervical cancer tissues and cervical cancer cell lines

**Fig. 2:**
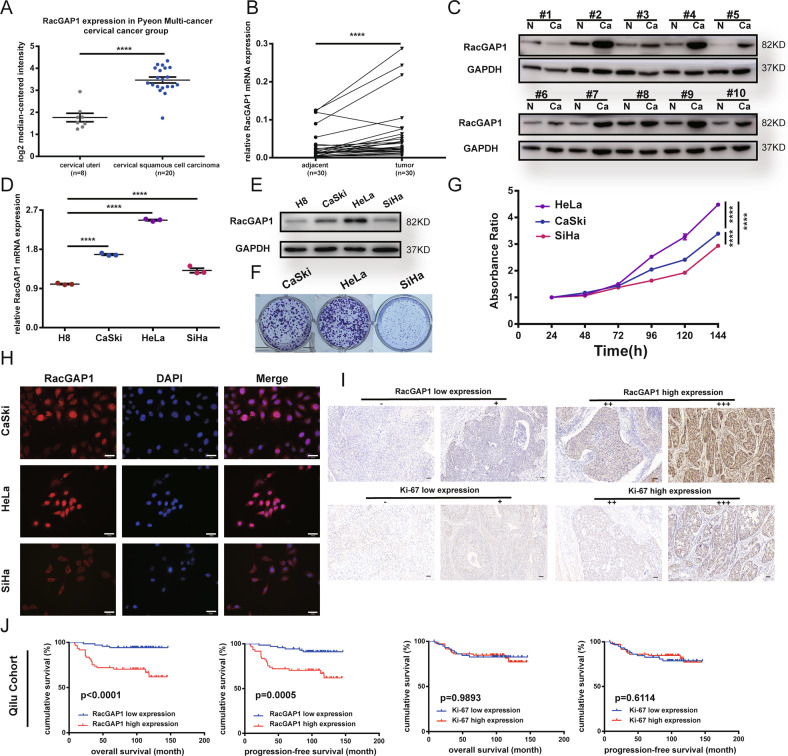
Expression of RacGAP1 in specimens of cervical cancer patients and cell lines. **A** RacGAP1 mRNA was overexpressed in Pyeon Multi-cancer dataset from Oncomine database. **B** Expression of RacGAP1 mRNA in 30 cervical cancer specimens and paired adjacent normal tissues by qRT-PCR. Wilcoxon test of paired *t* test, *p* < 0.0001. **C** Expression of RacGAP1 protein in 64 cervical cancer tissues and adjacent tissues was analyzed by Western blot, using GAPDH as an internal control. **D**, **E** Analyses of mRNA and protein expression levels of RacGAP1 in cervical cancer cell lines compared with normal cervical epithelium cell H8. **F** Clone formation assay of three cervical cancer cell lines. **G** Cell proliferation was detected by CCK8 assays. **H** Immunofluorescence staining showed that RacGAP1 protein was expressed in three cervical cancer cell lines and was localized in both cytoplasm and nuclei. Scale bar = 20 μm. **I** Representative RacGAP1 and Ki67 immunohistochemical staining in paraffin-embedded human cervical cancer tissues. (Magnification, ×400). **J** Kaplan–Meier survival analysis (log-rank test) of the correlation between RacGAP1/Ki67 expression and OS/PFS in 131 cervical cancer patients. GAPDH glyceraldehyde 3-phosphate dehydrogenase, RacGAP1 Rac GTPase-Activating Protein 1, qRT-PCR quantitative real-time polymerase chain reaction, OS overall survival, PFS progression‐free survival.

To explore the expression of RacGAP1 in CC, we analyzed a profile from the Oncomine database named Pyeon Multi-cancer. We found that RacGAP1 was overexpressed in cervical squamous cell carcinoma samples compared with normal cervix uteri epithelia (Fig. 2A). To validate this finding, we investigated RacGAP1 mRNA expression in 30 paired fresh tumor tissues and their paired adjacent noncancerous tissues by qRT-PCR and the protein expression in 64 paired CC and normal samples by Western blot. As shown in Fig. 2B, C, Fig. S1A, and Original Data 1A, RacGAP1 was significantly overexpressed in CC tissues compared with adjacent noncancerous tissues in mRNA and protein levels (*p* < 0.001). Among three CC cell lines, the RacGAP1 expression decreased sequentially in HeLa, CaSki, and SiHa (Fig. 2D, E, Fig. S1B, and Original Data 1B) and the proliferation ability also decreased in turn (Fig. 2F–G and Fig. S1 D). RacGAP1 is localized in the nuclei and the cytoplasm of three cell lines (Fig. 2H).

### The correlation of RacGAP1 with clinicopathological parameters and prognostic significance

To investigate the clinical significance of RacGAP1 in CC, we detected the expression of RacGAP1 by IHC staining in a retrospective cohort of 131 CC specimens. As shown in Fig. 2I, the expression of RacGAP1 was defined as yellow-brown staining of nuclei or cytoplasm. Table 1 summarized the correlation between the expression of RacGAP1 and the clinicopathological parameters. The results indicated that the expression of RacGAP1 was significantly correlated with the histological grade (*p* < 0.001). RacGAP1 was highly expressed in poorly differentiated CC. The correlations between the expression and other parameters including age, histological type, FIGO stage, lymphatic metastasis, invasive interstitial depth, parametrium metastasis, LVSI or tumor size were not statistically significant. We also detected the expression of Ki67, which was also related to proliferation and differentiation (Fig. 2I). The Kaplan–Meier survival curves illustrated that the overall survival (OS) and progression-free survival (PFS) of CC patients with high expression levels of RacGAP1 (*n* = 61) were significantly shorter than those with low levels (*n* = 70) (Fig. 2J; *p* < 0.0001, *p* = 0.0005). However, Ki67 expression did not affect CC patients’ OS and PFS (Fig. 2J). Univariate and multivariate Cox regression analysis confirmed that the high RacGAP1 expression could be an independent predictor of poor survival in cervical cancer (Table 2).

**Table 1:** Correlations between RacGAP1 expression and clinicopathologic characteristics of cervical cancer patients.

		RACGAP1 expression, *N* (%)			
Characteristics	Cases	Low expression	High expression	*p* value	Spearman correlation
Age					
≤50	90 (68.70)	48 (36.64)	42 (32.06)	0.972	−0.003
>50	41 (31.30)	22 (16.79)	19 (14.50)		
Histological type					
Squamous	114 (87.02)	60 (45.80)	54 (41.22)	0.633	−0.042
Nonsquamous	17 (12.98)	10 (7.63)	7 (5.34)		
Figo stage					
I	91 (69.47)	49 (37.40)	42 (32.06)	0.887	0.012
II~	40 (30.53)	21 (16.03)	19 (14.50)		
Histological grade					
Well/moderate	60 (45.80)	42 (32.06)	18 (13.74)	<0.001*	0.305
Poorly	71 (54.20)	28 (21.37)	43 (32.82)		
Lymphatic metastasis					
No	97 (74.05)	54 (41.22)	43 (32.82)	0.386	0.076
Yes	34 (25.95)	16 (12.21)	18 (13.74)		
Invasive interstitial depth					
<1/2	30 (22.90)	17 (12.98)	13 (9.92)	0.686	0.035
≥1/2	101 (77.10)	53 (40.46)	48 (36.64)		
Tumor size					
*d* ≤ 4 cm	98 (74.81)	56 (42.75)	42 (32.06)	0.143	0.128
*d* > 4 cm	33 (25.19)	14 (10.69)	19 (14.50)		
Parametrium metastasis					
No	127 (96.95)	67 (51.15)	60 (45.80)	0.712	−0.077
Yes	4 (3.05)	3 (2.29)	1 (0.76)		
LVSI					
No	115 (87.79)	62 (47.33)	53 (40.46)	0.769	0.026
Yes	16 (12.21)	8 (6.11)	8 (6.11)		

*FIGO* International Federation of Gynecology and Obstetrics, *LVSI* lymph-vascular space invasion, *RacGAP1* Rac GTPase-activating Protein 1.

**Table 2:** Univariate and multivariate Cox regression analyses on progression-free survival in cervical cancer.

	Univariate analysis			Multivariate analysis		
Variables	*p* value	HR	95% CI	*p* value	HR	95%CI
Age	0.738	0.861	0.359–2.064			
Histological type	0.078	2.285	0.911–5.733			
Figo stage	0.831	0.909	0.380–2.177			
Histological grade	0.756	1.134	0.514–2.498			
Tumor size	0.024	2.487	1.127–5.486			
LVSI	0.001	3.932	1.693–9.131			
LN	0.000019	5.782	2.589–12.914	0.000035	5.638	2.486–12.789
Invasive interstitial depth	0.938	0.964	0.385–2.415			
Parametrium metastasis	0.012	4.69	1.401–15.705	0.005	6.188	1.756–21.804
RACGAP1 expression	0.002	4.221	1.683-10.586	0.002	4.389	1.740–11.072

*FIGO* International Federation of Gynecology and Obstetrics, *LVSI* lymph-vascular space invasion, *RacGAP1* Rac GTPase-activating protein 1.

### The role of RacGAP1 in CC cell growth, migration, and invasion

**Fig. 3:**
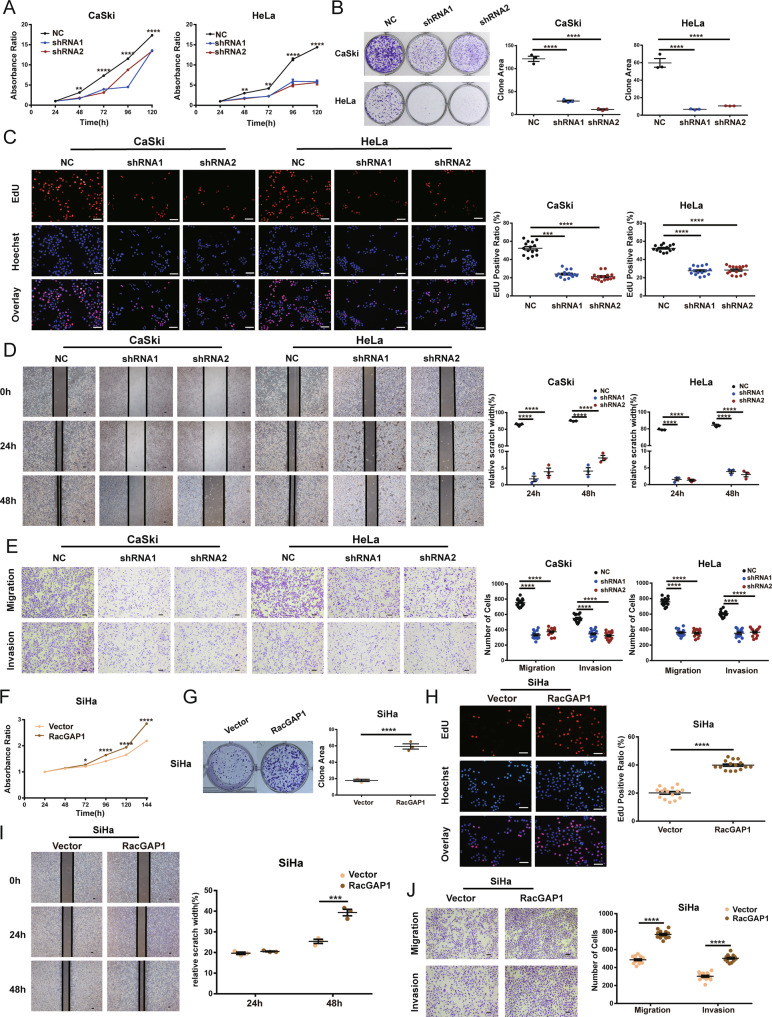
Effects of RacGAP1 on cell proliferation, invasion, and migration. **A**–**C** Cell proliferation was detected by CCK8 assays, clone formation assays, and EdU assays after RacGAP1 knockdown in CaSki and HeLa cell lines. **D**, **E** Wound healing assay and transwell assays showed downregulation of RacGAP1 reduces the ability of invasion and migration in CaSki and HeLa cells. Scale bar = 50 μm. **F**–**H** Cell proliferation was detected by CCK8 assays, clone formation assays, and EdU assays after RacGAP1 upregulated in SiHa cell line. **I**, **J** Wound healing assay and transwell assays showed upregulation of RacGAP1 promoted the ability of invasion and migration in SiHa cells. Scale bar = 50 μm. The data were presented as means ± SEMs, *p < 0.05, **p < 0.01, ***p < 0.005, ****p < 0.001; RacGAP1 Rac GTPase-activating Protein 1, EdU 5-ethynyl-2’ -deoxyuridine, SEM standard error of the mean.

CaSki and HeLa cells with the downregulated endogenous expression of RacGAP1 were established with lentiviruses carrying specific shRNAs. SiHa cells were transfected with lentiviral vectors containing the RacGAP1 sequence. The efficiency was confirmed using qRT-PCR and Western blot analyses (Fig. S1 C, E–G and Original Data 3B, C). Flag fusion protein was used to compare the exogenous and endogenous RacGAP1 proteins (Fig. S1 H and Original Data 3D). To further explore the role of RacGAP1 in regulating proliferation, migration, and invasion in CC cells, a series of molecular functional experiments were conducted. Growth curves detected by the CCK8 assay, clone formation assays, and EdU assays were performed to determine the RacGAP1 function in regulating proliferation. Compared with the NC group, knocking down RacGAP1 expression suppressed CC cell proliferation. (Fig. 3A–C). On the contrary, the proliferation of RacGAP1 overexpression cells was significantly increased (Fig. 3F–H). Wound healing assay and transwell assay were used to analyze the effects of RacGAP1 on migration and invasion of CC cells. The results indicated that downregulation of RacGAP1 decreased the migratory and invasive abilities of CaSki and HeLa cells (Fig. 3D, E). Consistent with this, upregulation of RacGAP1 resulted in a significant enhancement in migratory and invasive abilities of SiHa cells (Fig. 3I, J).

**Fig. 4:**
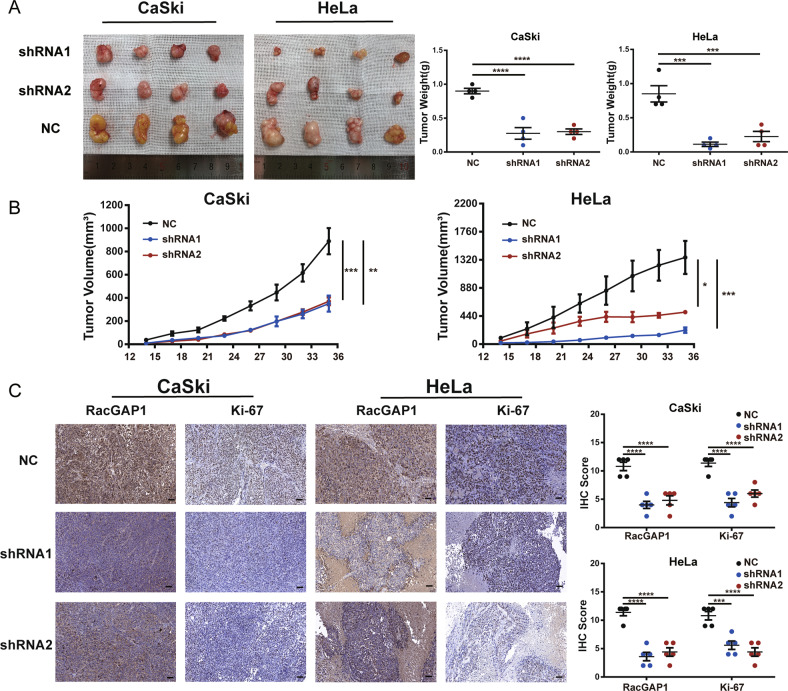
The effects of RacGAP1 expression on tumorigenesis in vivo. **A** Images and weights of xenograft tumors. **B** The growth curves of xenograft tumors. The volumes of tumors were monitored every 3 days. **C** Representative images of IHC staining of RacGAP1/Ki67 in tumor tissues (magnification, ×400). IHC scores of each group. Data are mean ± SEM, *p < 0.05, **p < 0.01, ***p < 0.005, ****p < 0.001, *n* = 4, RacGAP1 Rac GTPase-Activating Protein 1.

In vivo experiment was performed to further validate the importance of RacGAP1 in CC growth regulation. We monitored tumor growth by subcutaneously injecting KD and NC cells into nude mice. Results showed that the tumorigenicity of KD cells was significantly reduced compared with NC cells during the same period (Fig. 4A, B). Meanwhile, the IHC staining proved that the expression of Ki67 in the KD group was significantly lower than that in the control group (Fig. 4C). In summary, these results indicated that RacGAP1 played an important role in the tumorigenicity of CC cells.

### miR-192 was a downstream target of RacGAP1 in CC

**Fig. 5:**
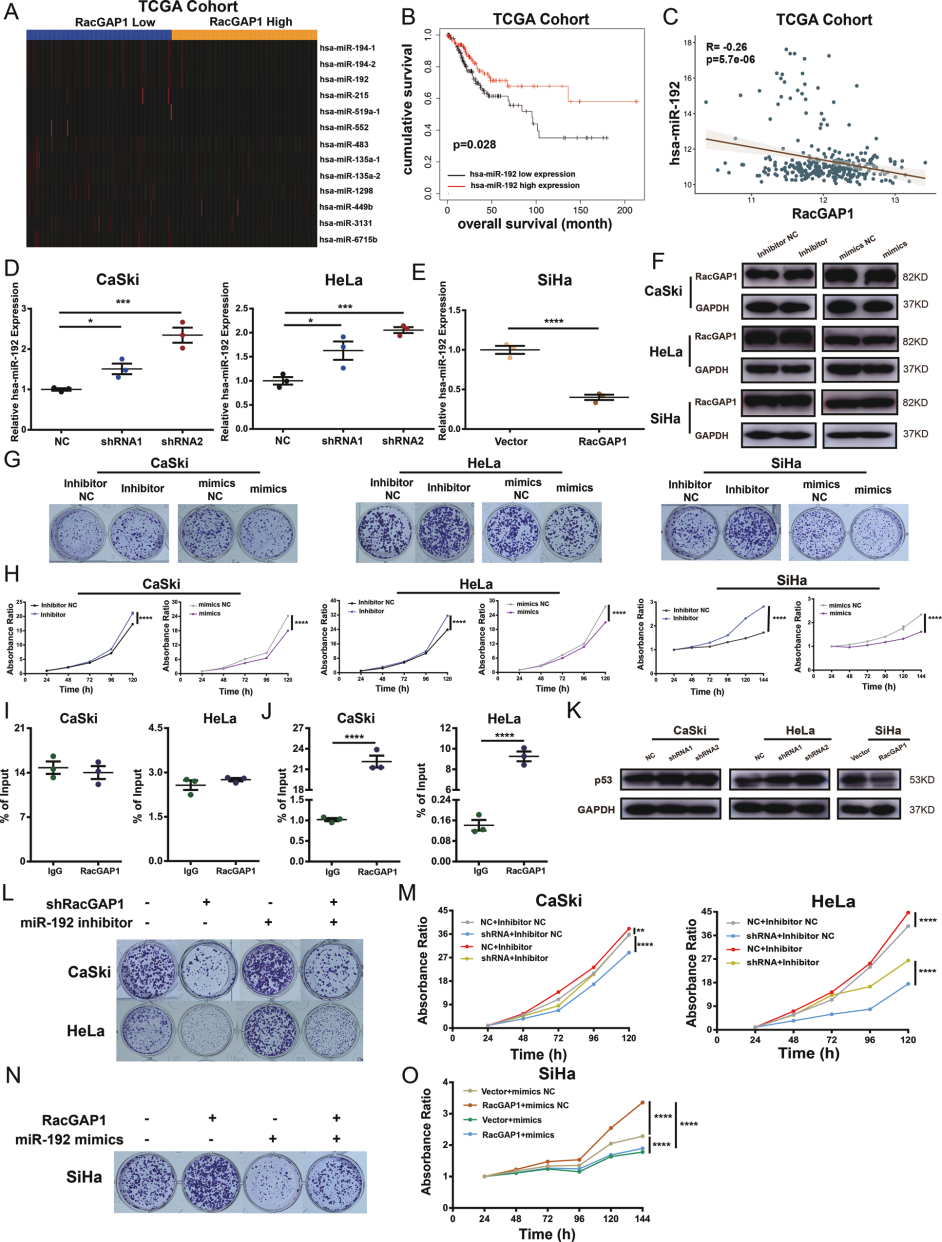
miR-192 was a downstream target of RacGAP1 which could affect cell proliferation, migration, and invasion. **A** Heatmap of differentially expressed miRNAs in low and high RacGAP1 expression CC tissues from TCGA. **B** Kaplan–Meier survival analysis (log-rank test) of the correlation between miR-192 expression and OS in cervical cancer patients from TCGA database. **C** miR-192 was negatively correlated with the expression of RacGAP1. *p* = 5.7e-08, *R* = −0.26. **D** The expression of miR-192 was upregulated in RacGAP1 knockdown CC cells. **E** The expression of miR-192 was downregulated in RacGAP1 overexpressed CC cells. **F** Expression of RacGAP1 in CC cells was not changed after transfected with miR-192 inhibitor or mimics. **G**, **H** Cell proliferation was detected by clone formation assays and CCK8 assays after being transfected with miR-192 inhibitor or mimics. **I** The interaction between RacGAP1 and miR-192 was validated by RIP-PCR in CaSki and HeLa cells (*n* = 3 biologically independent samples). **J** The interaction between RacGAP1 and TP53 mRNA was validated by RIP-PCR in CaSki and HeLa cells (*n* = 3 biologically independent samples). **K** Knocking down RacGAP1 increased the expression of p53 protein while RacGAP1 overexpression suppressed p53 expression. **L**–**O** Clone formation assays and CCK8 assays of the rescue experiment in CC cells. The data were presented as means ± SEMs. *p < 0.05, **p < 0.01, ***p < 0.005, ****p < 0.001. TCGA The Cancer Genome Atlas, RacGAP1 Rac GTPase-activating protein 1.

We analyzed the miRNA-seq from TCGA using “edgeR” package. We divided the data into two groups according to the RacGAP1 expression level and acquired 13 differentially expressed miRNAs (Fig. 5A). Among these miRNAs, we found that the expression of miR-192 was negatively correlated with RacGAP1 expression and patients with high expression of miR-192 had a better prognosis (Fig. 5B, C, Fig. S1I). Knocking down RacGAP1 expression could elevate the expression of miR-192 while overexpressing RacGAP1 could decrease the miR-192 expression (Fig. 5D–E). Three CC cell lines were transfected with the miR-192 inhibitor and mimics. The efficiency was measured using qRT-PCR (Fig. S1J). Changing the miR-192 expression didn’t influence the RacGAP1 expression (Fig. 5F, Fig. S1L and Original Data 1C). CCK8 and clone formation assays showed that inhibiting miR-192 promoted the proliferation of CC cells while overexpressing miR-192 had opposite effects (Fig. 5G–H, Fig. S1K). Transwell assay and wound healing assay revealed that enhancing the expression of miR-192 decreased the migration and invasion ability of CC cells while decreasing expression was on the contrary (Fig. S2).

The RIP-PCR results indicated that miR-192 expression had no statistical difference between groups of RacGAP1 pulldowns and IgG controls (Fig. 5I). As a transcriptional factor of mir-192, TP53 expression was significantly higher in RacGAP1 pulldowns than in IgG controls (Fig. 5J). Knocking down RacGAP1 increased the p53 protein expression while RacGAP1 overexpression had a contrary result (Fig. 5K, Fig. S1M, and Original Data 1D). These results suggest that the RacGAP1 regulated miR-192 via TP53.

Rescue experiments were performed to verify whether miR-192 was involved in RacGAP1 mediated malignant effects on CC cells. We knocked down RacGAP1 and miR-192 in CC cells simultaneously and discovered that inhibiting miR-192 rescinded the reduced cell proliferation, migration, and invasion induced by RacGAP1 knockdown (Fig. 5L, M, Fig. S3A, C). Meanwhile, amplifying miR-192 could reduce cell proliferation, migration, and invasion induced by RacGAP1 overexpression (Fig. 5N, O, Fig. S3B, D). These results suggested that miR-192 was involved in RacGAP1 mediated oncogenic behaviors of CC cells.

### RacGAP1 promoted malignant progression in CC cells by regulating AP-1 via miR-192 and p-JNK

**Fig. 6:**
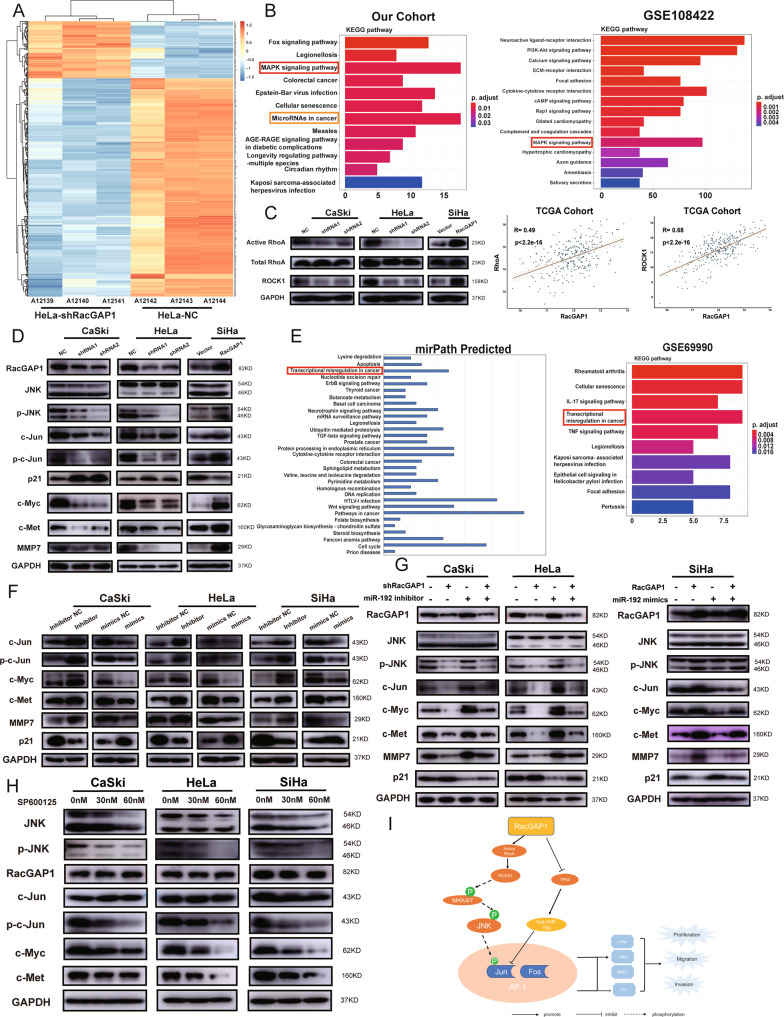
RacGAP1 activated AP-1 via miR-192 and p-JNK. **A** Microarray analysis of Hela cell RacGAP1 KD compared with NC. **B** KEGG pathway enrichment analyses of DEGs from our cohort and GSE108422. **C** Detection of RhoA activity and ROCK1 expression in RacGAP1 knockdown or -overexpressing CC cells. RacGAP1 was positively correlated with RhoA and ROCK1 (*R* = 0.49 and *R* = 0.68, *p* < 2.2e-16). **D** Changes in the expression of the genes after RacGAP1 knockdown or overexpressed in CC cells were detected by Western blot. **E** KEGG pathway enrichment analyses of miR-192 target genes and DEGs from GSE69990. **F** Western blot analysis was used to detect the protein expression after cells were transfected with miR-192 inhibitor and mimics. **G** Western blot analysis of the protein expression of the rescue experiment in CC cells. **H** Western blot analysis of the protein expression changes of CC cells treated with SP600125. **I** Pattern diagram of RacGAP1 effect to cervical cancer. RacGAP1 Rac GTPase-activating protein 1, JNK c-Jun N-terminal kinase, MMP7 matrix metallopeptidase 7, ROCK1 Rho-associated coiled-coil containing protein kinase 1. The data were presented as means ± SEM. *p < 0.05, **p < 0.01, ***p < 0.005, ****p < 0.001.

To further elucidate the mechanisms of RacGAP1 function, we performed microarray analysis and got 436 DEGs (Fig. 6A). The results of GO annotation were shown in Fig. S4A. KEGG pathway enrichment showed that 188 DEGs were significantly enriched in 12 KEGG pathways. The most abundant genes (18 genes) were distributed in the MAPK signaling pathway, followed by microRNAs in cancer. Meanwhile, DEGs from GSE108422 dataset were also enriched in MAPK signaling pathway (Fig. 6B). RacGAP1 is a Rho GTPase-activating protein. Active GTPase Immunoprecipitation showed that RacGAP1 could increase the level of active RhoA, which in turn caused changes in the downstream effector ROCK1. RacGAP1 was positively correlated with RhoA and ROCK1 (Fig. 6C, Fig. S4 B, and Original Data 1E). Western blot analysis showed that knocking down RacGAP1 inhibited JNK phosphorylation while overexpressing RacGAP1 increased the expression of phosphor-JNK (p-JNK). JNK, Erk, phosphor-Erk (p-Erk), p38, and phosphor-p38 (p-p38) in these groups were not changed. Phosphor-MKK4 (p-MKK4) and phosphor-MKK7 (p-MKK7), as an upstream factors regulating JNK phosphorylation, were also corresponding changed. Furthermore, knocking down RacGAP1 downregulated the expression of c-Jun, which is an important component of AP-1. Downregulation of phosphor-c-Jun (p-c-Jun), c-Myc, c-Met, MMP7, and upregulation of p21 were also observed. Upregulation of RacGAP1 had the opposite results (Fig. 6D, Fig. S4C–E, Original Data 1F and 4A). p-JNK, c-Jun, p-c-Jun, and c-Myc were also tested in the xenograft tumor model by IHC. The results were similar to the western blot results (Fig. S5 A).

KEGG pathway enrichment of miR-192 target genes got “transcriptional misregulation in cancer”, which was also shown in the result from the GSE69990 (Fig. 6E). Western blot analysis showed that dysregulation of miR-192 did not affect the expression of JNK, p-JNK (Fig. S4 F and Original Data 4B). The expression of c-Jun, p-c-Jun, c-Myc, c-Met and MMP7 were upregulated when miR-192 was inhibited while the results were opposite when downregulating miR-192. However, normalizing with c-Jun, changes in p-c-Jun were not statistically different between groups suggesting that miR-192 affected the expression of c-Jun rather than its phosphorylation. The result of p21 was opposite to the result of c-Jun. (Fig. 6F, Fig. S4 G, H and Original Data 2A).

Rescue experiments were performed to investigate whether RacGAP1 activated the AP-1 through miR-192. Western blot showed that the protein expression of c-Jun and the downstream genes, which was downregulated by RacGAP1 knockdown, were enhanced after miR-192 was inhibited. Moreover, the protein expression of c-Jun and the downstream genes, which was upregulated by RacGAP1 overexpression, were decreased after miR-192 was amplified. However, the expression of RacGAP1, JNK and p-JNK was not changed (Fig. 6G, Fig. S5 B and Original Data 2B).

To confirm that dysregulation of miR-192 and c-Jun wasn’t caused by JNK phosphorylation, we used JNK inhibitor, SP600125, to treat CC cell lines. SP600125 could inhibit cell proliferation (Fig. S5 D). The miR-192 expression was not change after treated with SP600125 (Fig. S5 E). Western blot showed that the expression of c-Jun was not affected after treated with SP600125, but JNK, p-JNK, p-c-Jun, c-Myc and c-Met were downregulated (Fig. 6H, Fig. S5F and Original Data 3A). All these results indicated that RacGAP1 promoted malignant progression in CC cells by regulating AP-1 via miR-192 and p-JNK (Fig. 6I).

## Discussion

The occurrence of CC is a complex process, and most cervical malignancies are associated with high-risk HPV infection and other non-viral factors. Some studies suggest that oncogenes are directly related to tumorigenesis and therefore may be potential therapeutic targets. In our study, bioinformatics analysis showed that RacGAP1 was a key candidate gene in the progression of CC.

Previous studies showed that elevated expression of RacGAP1 has been observed in several human cancers including breast cancer, melanoma, hepatocellular cancer, gastric cancer, head and neck squamous cell cancer (HNSCC), meningioma, etc. and linked with poor prognosis [11–15]. RacGAP1 could be a predictive biomarker for lymph node metastasis and poor prognosis in colorectal cancer [21], but Yeh CM et al. found that according to the expression position of RacGAP1 in colorectal cancer cells, patients had the opposite prognosis [22]. Besides the predictive role of RacGAP1 in cancers, it may also be a new target in developing novel chemotherapy drugs. RacGAP1-dependent activation of AKT mediated doxorubicin resistance in HNSCC cells. RacGAP1 downregulation in HNSCC cells showed slower growth and more sensitive to doxorubicin in mice models [19]. In our study, we found that RacGAP1 was upregulated in cancer tissues and correlated with histological grade. High expression of RacGAP1 was associated with poor prognosis and was an independent prognostic factor for CC. To confirm our bioinformatics analyses and clinical findings, molecular functional experiments were conducted and the results suggested RacGAP1 was involved in the proliferation, migration, and invasion, which served as an oncogene in CC both in vitro and in vivo. And it may be a potential therapeutic target for cervical cancer patients.

Yukio Tonozuka et al. found that RacGAP1 enhanced IL-6-induced differentiation through enhancement of STAT3 activation [23]. Similarly, work by Mi S et al. suggested that RacGAP1 promoted cell motility and invasion by regulating STAT3 phosphorylation and survivin expression [24]. RacGAP1-depleted cells failed to proliferate as the result of the CDK inhibitor CDKN1A/p21 upregulated and caused the onset of the senescence [25]. RacGAP1 promoted the activations of RhoA, FAK, paxillin and triggered focal adhesion formation and cytoskeletal rearrangement. Zhang et al. suggested RacGAP1 mediated endothelial barrier function loss and melanoma transmigration in a focal adhesion-dependent manner. What's more, the expression of RacGAP1 in endothelial cells may play a key role in the pathogenesis of cancer by regulating endothelial permeability [15]. In our study, miR-192 was identified as the downstream of RacGAP1 which was involved in RacGAP1 mediated malignant effects on CC cells.

Previous study showed that hsa-miR-192 was a tumor suppressor. It could suppress cell proliferation, metastasis, and stemness and induce apoptosis in different cancer types [26–32]. But its function in CC remained unknown. In our study, it was found that miR-192 was negatively correlated with RacGAP1 and suppressed tumor malignant progression. Inhibition of miR-192 could rescue the proliferation, migration, and invasion suppression caused by knocking down RacGAP1. Although RIP-PCR results showed that RacGAP1 cannot directly target miR-192, RacGAP1 could bind to TP53 mRNA directly and negatively regulate p53 protein expression. A previous study showed that TP53, as a transcriptional factor, could activate miR-192 expression [33–36]. So, RacGAP1 may regulate miR-192 expression via TP53.

RacGAP1, as a Rho GTPase-activating protein, binds activated forms of Rho GTPases and stimulates GTP hydrolysis. However, more and more studies have shown that RacGAP1 played an important role in mediating a rapid cycling between GTP-RhoA and GDP-RhoA (GTPase flux), which could maintain a focused RhoA activity zone and increase RhoA activity [37–41]. In our study, RacGAP1 could increase the level of active RhoA, which in turn caused changes in the downstream effector ROCK1 in cervical cancer.

To explore the possible mechanism in cervical cancer, microarray analysis was carried out and the results showed a downregulation of c-Jun in RacGAP1 KD cells compared with NC cells. Western blot validated that downregulation of RacGAP1 decreased the expression of c-Jun, p-c-Jun while upregulation RacGAP1 had the opposite result.

c-Jun is the most important component of AP-1 (activating protein 1). AP-1 transcription factor is a dimeric transcription factor encompassing a group of structurally and functionally related members of c-Jun, c-Fos, ATF, and MAF protein families [42–44] and it can therefore form many different combinations of heterodimers and homodimers, and this combination determines the genes that are regulated by AP-1 [45]. AP-1 can regulate a wide range of biological processes including proliferation, differentiation, apoptosis, survival, migration, invasion, and transformation [42, 44, 46–50]. The activation of the AP-1 is regulated at two major levels: extracellular stimuli modulate both the abundance and the activity of AP-1 proteins. The abundance of AP-1 proteins is most commonly regulated by controlling the transcription of their genes and modulating their stability [46]. Phosphorylation of c-Jun by JNK on serine 63 and 73 could increases its stability to a certain extent and increase its transcriptional activity [51, 52]. There is evidence that AP-1 formation proteins, especially Jun group proteins, control cell proliferation, migration and invasion through their ability to regulate the expression and function of many genes such as Cyclin D1, p21^cip1/waf1^, p19^ARF^, p16, c-Myc, β-catenin, matrix metalloproteinases(MMPs) and VEGFA [53–59]. In our study, Western blot validated that RacGAP1 could influence the expression of c-Jun via miR-192, and phosphorylation of c-Jun via p-JNK separately. Changing in miR-192 expression did not affect the phosphorylation of c-Jun. Downregulation of p-JNK did not affect the expression of c-Jun. This could influence the activation of AP-1 and regulate the downstream genes such as c-Myc, c-Met, p21 and MMP7 to affect cell proliferation, migration, and invasion. Though miR-192 could not directly target c-Jun, it could directly target RB1 and suppressed its expression [60–62]. RB1 could be a transcriptional activator binding to c-Jun and activating its transcription [63, 64]. The specific mechanism is one of our future research directions.

In summary, our findings demonstrated the potential role of RacGAP1 in the progression of CC. RacGAP1 was an independent factor for poor prognosis. We also found that RacGAP1 regulated the expression of c-Jun via miR-192 and phosphorylation of c-Jun via p-JNK separately to activate AP-1. This could promote cancer cell proliferation, migration, and invasion. RacGAP1 could be considered a potential therapeutic target in future treatment development.

### Supplementary information

Supplementary Material 1

Supplementary Material 2

Table S1

Figure S1

Figure S2

Figure S3

Figure S4

Figure S5

Original Data 1

Original Data 2

Original Data 3

Original Data 4

Figure Legend for Supplementary Figures

Reproducibility checklist

all of the co-authors’ email responses to the author's addition

## Data Availability

The data sets used and/or analyzed during the current study are available from the corresponding author on reasonable request.
