# Correction: RacGAP1 promotes the malignant progression of cervical cancer by regulating AP-1 via miR-192 and p-JNK

**DOI:** 10.1038/s41419-024-06880-7

**Published:** 2024-07-24

**Authors:** Tianli Zhang, Chunyan Wang, Kun Wang, Ying Liang, Ting Liu, Liping Feng, Xingsheng Yang

**Affiliations:** 1 Department of Obstetrics and Gynecology, Qilu Hospital of Shandong University, Jinan, Shandong 250012 People’s Republic of China; 2 Key Laboratory of Gynecologic Oncology of Shandong Province, Qilu Hospital of Shandong University, Jinan, Shandong 250012 People’s Republic of China

**Keywords:** Cervical cancer, Cervical cancer

Correction to: *Cell Death and Disease* 10.1038/s41419-022-05036-9, published online 12 July 2022

The original version of this article unfortunately contained errors in Figure 5G. The clone formation pictures of CaSki cell treated with Inhibitor NC and Inhibitor were misused. They were misused as the same pictures of CaSki cells treated with mimics NC and mimics. According to the raw data, this mistake didn’t influence the conclusion. The authors apologize for the mistake. The corrected figures can be found below.
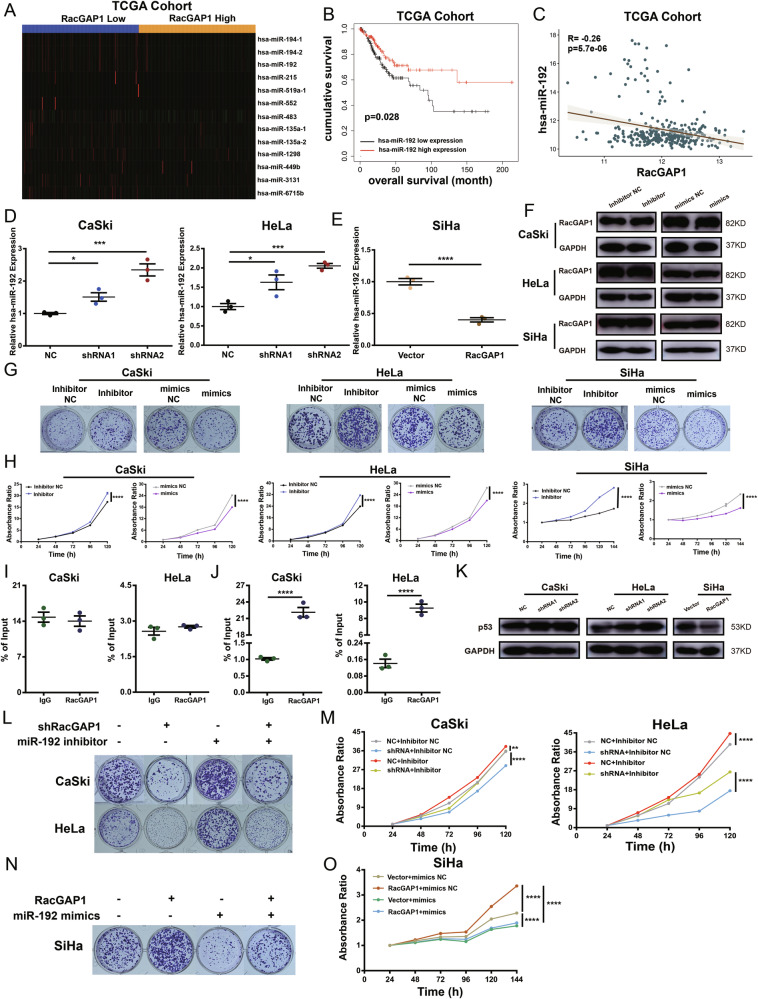

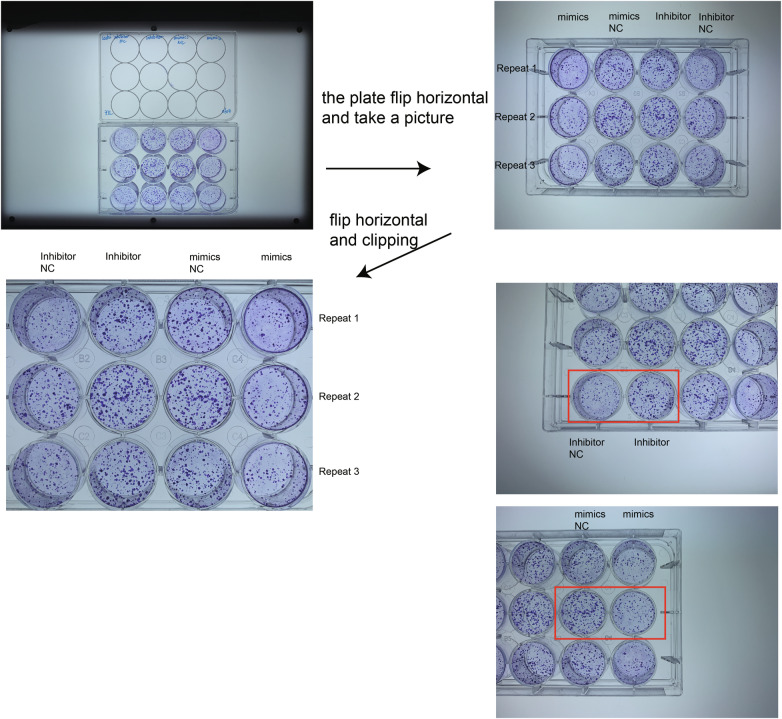


The original article has been corrected.

