# Supplementary material for: RacGAP1 promotes the malignant progression of cervical cancer by regulating AP-1 via miR-192 and p-JNK
Source: Cell Death Dis. 2022 Jul 12;13(7):604. doi: 10.1038/s41419-022-05036-9 (PMC9279451; doi:10.1038/s41419-022-05036-9)
Supplement: Supplementary file 1 — Supplementary Material 1 [file 41419_2022_5036_MOESM1_ESM.docx]

**Supplementary Material 1**

**Materials and Methods**

**Antibodies and Reagents**

RacGAP1 (NBP1-33455), Novus Biologicals Inc., Littleton, CO, USA; c-Met (ab51067), Ki-67 (ab15508), p53 (ab26), Abcam, Cambridge, UK; GAPDH (2118), Erk1/2 (4695), p-Erk1/2 (4370), p38 (8690), p-p38 (4511), p21 (2947), c-Jun (60A8), p-c-Jun (D47G9), ROCK1 (4035), MKK4 (9152), p-MKK4 (9156), MKK7 (4172), p-MKK7 (4171), CST, Danvers, MA, USA; JNK (66210-1-Ig), c-Myc (10828-1-AP), Proteintech Group Inc, Rosemont, IL, USA; p-JNK (AF3318), MMP7(AF0218), Affinity Biosciences LTD, Jiangsu, China.

SP600125 (A4604), APExBIO Technology LLC, Houston, TX, USA.

**Bioinformatics analyses**

The gene expression profiles of GSE7803, GSE9750, GSE63514, GSE56363, GSE70035, GSE108422 and GSE69990 were obtained from the National Center of Biotechnology Information GEO database (GEO, <http://www.ncbi.nlm.nih.gov/geo/>). Details were shown in Table S1. miRNA-seq data of cervical squamous cell carcinoma and endocervical adenocarcinoma (CESC) was downloaded from The Cancer Genome Atlas (TCGA). Genes meeting the criterion of |log_2_Foldchange| > 1 and p-value < 0.05 were identified as differentially expressed genes (DEGs). For microarray data, we used the R package “limma” to normalize the data and find DEGs, and Robust Rank Aggregation (RRA) was used to find the most significant DEGs of GSE7803, GSE9750, and GSE63514. For the TCGA data, we used “edgeR” R package. DEGs of tissues and SiHa cells with or without HPV16 E6/E7 were obtained from supplementary materials of *Transcriptome sequencing profiles of cervical cancer tissues and SiHa cells* submitted by Tingting Chen et al. Finally, the overlap of the gene sets was shown by Venn diagram.

We conducted Gene Ontology (GO) enrichment and Kyoto Encyclopedia of Genes and Genomes (KEGG) pathway analyses using the R package “clusterprofiler”. To identify interaction functions among DEGs, the genes were entered into Search Tool for the Retrieval of Interacting Genes (STRING) for further analyses and visualized using Cytoscape plugin. Next, we conducted module analyses via the cytoHubba plugin to identify the top 10 nodes ranked by MCC. Genes with adjusted p<0.05 were defined as significant difference.

We used the Oncomine Platform (<http://www.oncomine.org>) to further confirm the RacGAP1 expression in CC. The RacGAP1 mRNA expression data of each array was logarithmically transformed, centered on median, and standardized standard error of mean (SEM).

Target genes of hsa-miR-192 were obtained from DIANA TOOLS (<http://diana.imis.athena-innovation.gr/DianaTools/index.php> ) which provided algorithms, databases and software for the annotation of miRNA targets such as microT-CDS, TarBase and TargetScan.

**Human cervical cancer specimens**

Fresh tissues were stored in liquid nitrogen before use. Patients with autoimmune disease, cancer in other systems, or received preoperative chemotherapy or radiotherapy were excluded from this study. Complete follow-up information was obtained for all paraffin-embedded samples.

**Immunohistochemistry and immunohistochemical analysis**

The 4-mm-thick paraffin-embedded tissue sections were deparaffined with xylene and rehydrated in an ethanol gradient. Then we used microwave and Tris-EDTA buffer (pH=9.0) or Sodium citrate buffer (pH=6.0) for heat-induced epitope retrieval. These specimens were dyed according to the manufacturer’s protocol. Sections were incubated with specific anti-RacGAP1 (1:100), anti-Ki67 (1:600), p-JNK (1:150), c-Jun (1:150), p-c-Jun ( 1:100) and c-Myc (1:200) antibodies at 4°C, overnight. After rinsing in phosphate-buffered saline (PBS), the sections were incubated with reagents 2 and 3 (PV-9001; Zhong Shan Biotech Co Ltd, Beijing, China) respectively. Subsequently, 3,3′-diaminobenzidine was added for visualization and 10% Mayer’s hematoxylin was used as counter stain. PBS was used as a negative control. The staining intensity was evaluated according to the following criteria: 0 (negative), 1 (low), 2 (medium), 3 (high). And the degree of staining was graded as 0 (<10%), 1 (11%-25%), 2 (26%-50%), 3 (51%-75%), 4 (76%-100%). The immunoreactive score (IRS) was defined as an intensity score multiplied by a degree score. IRS was further classified as − (0-3), + (3-6), ++ (6-9) and +++ (9-12). Median, 6.0 was set as the cut-off to distinguish the RacGAP1 expression low or high.

**RNA extraction and real-time polymerase chain reaction**

Total RNA was isolated from 31 paired fresh tissue specimens and cell lines using TRIzol (15596018; Invitrogen, Thermo Fisher Scientific Inc, Waltham, MA, USA) according to the manufacturers’ instructions. After measuring the concentrations of mRNA by a NanoDrop One Microvolume UV-Vis Spectrophotometer (Thermo Fisher Scientific Inc, Waltham, MA, USA), reverse-transcription kits (FSQ-301 and FSQ-101; Toyobo Biotech Co Ltd, Life Science Department, OSAKA, Japan) were used to synthesize complementary DNA. Quantitative reverse-transcription polymerase chain reaction (qRT-PCR) was performed using SYBR Green Real-time PCR Master Mix (QPK-201; Toyobo Biotech Co Ltd Life Science Department, OSAKA, Japan). Relative mRNA expression was calculated by comparative Ct method, and expression data were normalized to glyceraldehyde 3-phosphate dehydrogenase (GAPDH) or U6 snRNA to control variation. All the experiments were carried out in triplicates. The primer sequences were shown in Supplementary Material 2.

**Western Blot analysis**

RIPA (Beyotime, Shanghai, China) supplemented with PMSF was used to extract total protein from tissues or harvested cells. Equivalent amounts of proteins were separated by sodium dodecyl sulfate polyacrylamide gel electrophoresis and then transferred to polyvinylidene difluoride membranes. After being blocked with 5% fat-free milk in Tris-buffered saline (TBS) for 1 hour at room temperature (RT), the membranes were incubated with primary antibodies against RacGAP1 (1:1000), c-Met (1:1000), GAPDH (1:2000), p53 (1:1000), ROCK1 (1:1000), MKK4 (1:1000), p-MKK4 (1:1000), MKK7 (1:1000), p-MKK7 (1:1000), Erk1/2 (1:1000), p-Erk1/2 (1:1000), p38 (1:1000), p-p38 (1:1000), p21 (1:1000), c-Myc (1:1000), JNK (1:1000), p-JNK (1:500), c-Jun (1:1000), p-c-Jun (1:1000), MMP7(1:500) at 4°C, overnight. Subsequently, the membranes were incubated with horseradish peroxidase–conjugated anti-rabbit (7074) or anti-mouse (7076) secondary antibody (1:5000; Cell Signaling Technology, Inc, Danvers, MA, USA) for 1.5 hours at RT. ECL detection system (Amersham Imager 600; General Electric, Boston, USA) was used to visualize the results.

**Cell lines and cell culture**

Three HPV-positive human cervical cancer cell lines, HeLa, CaSki, SiHa and one cervical epithelial immortalized cell line, H8, were obtained from the Key Laboratory of Gynecologic Oncology of Shandong Province and cultured in Roswell Park Memorial Institute (RPMI) 1640 medium (C11875500BT, Gibco, Thermo Fisher Biochemical Products Co, Ltd, Beijing, China) supplemented with 10% fetal bovine serum (FBS; BI, 04-001-1 A; Biological Industries, Kibbutz Beit-Haemek, Israel) and 1% penicillin-streptomycin (15140-122, Gibco, Thermo Fisher Biochemical Products Co, Ltd, Beijing, China). All cells were maintained in a sterile humidified incubator at 37°C and 5% CO_2_. All cell lines were

confirmed using unique short tandem repeat (STR) analyses.

**Immunofluorescence staining**

CC cells seeded on coverslips (Nest, 801010: NEST Biotechnology Co, Ltd, Wuxi, Jiangsu, China) were fixed with 4% paraformaldehyde for 30 minutes when they reached a confluence of 60% to 70%. Cells were washed with cold PBS for three times and subsequently permeabilized with 0.5% Triton X-100 (T8200, Solarbio Science & Technology Co., Ltd., Beijing, China) for 10 minutes at RT. After blocking with normal goat’s serum for 30 minutes at RT, cells were incubated with primary antibodies against RacGAP1 (1:100) at 4°C, overnight. After thorough washing with PBS, cells were stained with DyLight594 conjugate goat anti-rabbit immunoglobulin G (IgG; 1:50; A23420; Abbkine Scientific Co., Ltd., Redlands, CA, USA) for 1 hour at RT in the dark. Cell nuclei were stained with 4′,6-diamidino-2-phenylindole (DAPI; AR1176; Boster Biological Technology Co Ltd, Wuhan, China) for 5 minutes. Immunofluorescence images were obtained by a fluorescence microscope (DP72; Olympus, Tokyo, Japan).

**Generation of stable gene knockdown cells by lentivirus transduction**

Short hairpin RNA (shRNA)-containing lentivirus, lentiviral particles containing amplified human RacGAP1 sequence lentiviral vectors and the corresponding negative control (NC) were designed and synthesized by GeneChem Co., Ltd., Shanghai, China to knock down or overexpress endogenous RacGAP1 expression. Lentivirus particles were transfected into the CC cells in the presence of polybrene and screened with 3 μg/mL puromycin for a week. The efficiency of RacGAP1 knockdown or upregulation was identified by qRT-PCR and Western blot analyses. The sequences were shown in Supplementary Material 2.

**Cell transfection**

After the cells reached 50% confluence in 6-well plates, the miR-192 inhibitor, mimics and the corresponding non‑specific miR control (inhibitor-NC, mimics-NC) synthesized by the GenePharma (Shanghai, China) were transfected into the cells using jetPRIME transfection reagent (Polyplus-transfection, New York, NY, USA) according to the manufacturer’s protocol. After 48h of transfection, the efficiency was detected through qRT-PCR. The sequences were shown in Supplementary Material 2.

***In vitro* proliferation assay**

Cell proliferation was evaluated using the Cell Counting Kit-8 (CCK-8; Dojindo Laboratories, Kumamoto, Japan), 5-ethynyl-2’-deoxyuridine (EdU, Cell-Light™ EdU Imaging Detecting Kit, RiboBio, Guangzhou, China) incorporation and clone formation assay.

For CCK-8 assay, CC cells were seeded in 96-well plates at a density of 1000 cells per well. Then, 10 μL of CCK-8 dissolved in serum-free RPMI 1640 was added to each well and incubated for 1 hours at 37°C followed by absorbance measurement at 450 nm wavelength. The optical density (OD) was measured once a day at the same time.

For EdU incorporation, CC cells were seeded on coverslips and after achieving 60% confluency on the coverslips, we followed the manufacturer’s protocol for the experiment. Photographs of the cells were captured using a fluorescence microscope at 5 random fields per coverslip. The ratio of EdU-positive nuclei to total nuclei was calculated as the proliferation rate.

For clone formation assay, CC cells were seeded in 12-well plates at a density of 1000 cells per well. One week later, viable colonies >0.1 mm in diameter were fixed and stained for 1 hour using 0.5% crystal violet diluted with 100% methanol.

Data are presented as the mean ± SEM. All the experiments were repeated three times.

**Cell migration and Matrigel invasion assays**

Migration and invasion of CC cells were assessed with 24-well transwell chambers (3422; Corning Incorporate, NY, USA) as previously reported. For invasion assay, the chambers were coated with 60 μL diluted Matrigel (1:8; 356234; Corning Incorporate, NY, USA) to create an artificial basement membrane. 600 μL RPMI 1640 medium containing 10% FBS was added into the lower chamber. Cells stably knocked down RacGAP1 were suspended in 200 μL FBS-free 1640 medium and added into the upper chambers (1.0×10^5^ cells/chamber) and then cultured for 24 hours (migration) or 36 hours (invasion) in an incubator. Afterwards, cells invaded to the bottom of the membrane were fixed with 100% methanol at RT for 30 minutes and stained with 0.5% crystal violet for 30 minutes. At least 5 random fields were used to quantify the number of invading cells under a light microscope. The experiments were repeated three times for each group.

**Wound healing assay**

After achieving 90% confluency in 6-well plates, the cells were cultured in serum-free medium for 24 hours and wounded on the monolayer of cells with a sterile pipette tip. Then, floating cells were removed with PBS and cultured again in serum-free 1640 medium. Photographs were taken at 0, 24, and 48 hours along the scrape line by microscope. Results were expressed as relative scratch width, based on the distance migrated relative to the original scratched distance. The experiment was carried out in triplicate under the same conditions.

**HeLa cell sequencing**

Total RNA of stable knocked down (KD) RacGAP1 and NC HeLa cells was extracted using TRIzol reagent following the manufacturer’s instructions. Microarray analysis was performed using PrimeView Human Gene Expression Array by GeneChem Co., Ltd., Shanghai, China.

***In vivo* tumorigenesis**

Female BALB/c nude mice (4‐5 weeks old, 12-16 g) were purchased from Nanjing GemPharmatech Laboratory (GemPharmatech Co., Ltd., Nanjing, China). The animals were housed in a specific pathogen-free environment. The laboratory animal room had designated asepsis chambers where were under constant conditions (20°C‐25°C, 50%‐65% humidity), and sterile food and water could be provided at will.

Mice were subcutaneously injected with 1.5 × 10^7^ HeLa cells and CaSki cells, NC or KD, respectively (4 mice for each group, randomly). When the implanted tumors were visible (about 14 days), we measured their length (L) and width (W) with a digital caliper every 3 days. Tumor volume was calculated as V = 0.5 × largest diameter × smallest diameter^2^. In the end (3 weeks later), the tumors were removed, photographed, and weighed. No blinding was done. All operations followed the national laboratory animal use and care guidelines. The animal study was reviewed and approved by the ethics committee of Cheeloo College of Medicine of Shandong University (21003).

**RNA immunoprecipitation (RIP)**

To analyze RNA molecules binding with RacGAP1, Magna RIP™ RNA-Binding Protein Immunoprecipitation Kit ( 17-700, Millipore, USA) was used to perform an RNA immunoprecipitation assay (RIP) according to the manufacturer’s protocol. Both input and RIP samples were prepared for qRT–PCR analysis as described in this section.

**Active GTPase Immunoprecipitation**

Immunoprecipitation of active RhoA was performed with the RhoA Activation Assay Kit (80601, NewEast Biosciences, USA) according to the manufacturer’s protocol. Briefly, when the density of CC cells reached about 80–90% confluence, cells were harvested using ice-cold 1X Assay/Lysis Buffer. Total protein concentration was measured using BCA method. Aliquot 1 mg of total cellular protein to a microcentrifuge tube and adjust the volume of each sample to 1 mL with 1X Assay/Lysis Buffer. Then, 1 μl of anti-active RhoA monoclonal antibody and 20 µl of resuspended protein A/G Agarose beads was added into assay tubes. Incubate the tubes at 4℃ for 1 hour with gentle agitation. The beads were collected by centrifugation for 1 min at 5000 × g and were washed three times with 0.5 ml 1× Assay/Lysis buffer. The bead pellet was resuspended in 20 µl of 2× reducing SDS-PAGE sample buffer and boiled for 5 min. Active-RhoA expression was detected by Western Blot using anti-RhoA antibody provided by the kit.

All cells have enough frozen stocks. All assays were performed using the cell lines within more or less similar passage numbers. Experiments would not be performed using cell lines beyond passage 30.
