# Supplementary material for: RacGAP1 promotes the malignant progression of cervical cancer by regulating AP-1 via miR-192 and p-JNK
Source: Cell Death Dis. 2022 Jul 12;13(7):604. doi: 10.1038/s41419-022-05036-9 (PMC9279451; doi:10.1038/s41419-022-05036-9)
Supplement: Supplementary file 2 — Supplementary Material 2 [file 41419_2022_5036_MOESM2_ESM.docx]

**Supplementary Material 2**

The primers

RacGAP1： Forward 5′-ACCTCTTCTGACCTTTCGCC-3′

Reverse 5′-CTGAGCCACTCTCTGCAAGT-3′

TP53: Forward 5′- CCTCAGCATCTTATCCGAGTGG-3′

Reverse 5′- TGGATGGTGGTACAGTCAGAGC-3′

hsa-miR-192: reverse-transcription

5‘-GTCGTATCCAGTGCAGGGTCCGAGGTATTCGCACTGGATACGACGGCTGT-3’

Forward 5‘- tcggcgCTGACCTATGAATTG-3’

Reverse 5‘- GTCGTATCCAGTGCAGGGTCC-3’t

Gene target sequences

RacGAP1

NC: 5’-TTCTCCGAACGTGTCACGT-3’

shRNA1: 5’- ATGGAACCAGATTCATCAA-3’,

shRNA2: 5’-AAAGTTGCCTTGTCGTCCT-3’

hsa-miR-192

inhibitor NC 5’-CAGUACUUUUGUGUAGUACAA-3’

inhibitor 5‘-GGCUGUCAAUUCAUAGGUCAG-3’

mimics NC sense 5‘-UUCUCCGAACGUGUCACGUTT-3’

antisense 5‘-ACGUGACACGUUCGGAGAATT-3’

mimics sense 5’-CUGACCUAUGAAUUGACAGCC-3’

antisense 5’-CUGUCAAUUCAUAGGUCAGUU-3’
