# Supplementary material for: RacGAP1 promotes the malignant progression of cervical cancer by regulating AP-1 via miR-192 and p-JNK
Source: Cell Death Dis. 2022 Jul 12;13(7):604. doi: 10.1038/s41419-022-05036-9 (PMC9279451; doi:10.1038/s41419-022-05036-9)
Supplement: Supplementary file 3 — Table S1 [file 41419_2022_5036_MOESM3_ESM.docx]

**Table S1 Characteristics of the included datasets.**

| Dataset ID | Country | number of samples | GPL ID |  | number of rows per platform |
| --- | --- | --- | --- | --- | --- |
| GSE7803 | USA | 10N 21T | GPL96 |  | 22283 |
| GSE9750 | USA | 24N 33T | GPL96 |  | 22283 |
| GSE63514 | USA | 24N 28T | GPL570 |  | 54675 |
| GSE56363 | Romania | 12CR 9NCR | GPL4133 |  | 45220 |
| GSE70035 | Japan | 6R 6NR | GPL570 |  | 54675 |
| GSE108422 | China | 3 si-control  3 siRacGAP1 | GPL570 |  | 54675 |
| GSE69990 | USA | 3 control miR-192  3 miR-192 | GPL10558 |  | 48107 |

Notes: GSE, Gene Expression Omnibus Series; GPL, Gene Expression Omnibus Platform; T, tumor samples; N, paracancerous normal samples; CR, complete response; NCR, non-complete response; R, response to neoadjuvant chemotherapy; NR, no response to neoadjuvant chemotherapy
