# Supplementary material for: RacGAP1 promotes the malignant progression of cervical cancer by regulating AP-1 via miR-192 and p-JNK
Source: Cell Death Dis. 2022 Jul 12;13(7):604. doi: 10.1038/s41419-022-05036-9 (PMC9279451; doi:10.1038/s41419-022-05036-9)
Supplement: Supplementary file 13 — Figure Legend for Supplementary Figures [file 41419_2022_5036_MOESM13_ESM.docx]

**Figure S1 (A)** Scatter plot of relative expression of RacGAP1 protein in 64 cervical cancer tissues and adjacent tissues. **(B)** Scatter plot of relative expression of RacGAP1 protein in CC cell lines and normal cervical epithelium cell H8. **(D)** Scatter plot of clone formation assay of three CC cell lines. **(C, E)** Efficiency of RacGAP1 shRNAs in CaSki and HeLa cell lines. **(F, G)** Efficiency of RacGAP1 overexpression in SiHa cell line. **(H)** Flag fusion protein was tested to compare the exogenous and endogenous RacGAP1 proteins. **(I)** Kaplan-Meier survival analysis (log-rank test) of the correlation between the 10 differentially expressed miRNAs expression and OS in cervical cancer patients from TCGA database. **(J)** Efficiency of miR-192 inhibitor and mimics in three CC cell lines. **(K)** Scatter plot of clone formation assay of three CC cell lines transfected with miR-192 inhibitor or mimics. **(L)** Scatter plot of relative expression of RacGAP1 protein of three CC cell lines transfected with miR-192 inhibitor or mimics. **(M)** Scatter plot of p53 protein gray value analysis in CC cells after RacGAP1 knockdown or overexpressed. Data was presented as means ± SEM. *p<0.05, **p<0.01, ***p<0.005, ****p<0.001

**Figure S2 Transwell and wound healing assays of CaSki, HeLa and SiHa transfected with miR-192 inhibitor or mimics. (A)** Transwell assays. **(B)** Wound healing assays. Scale bar = 50μm. The data was presented as means ± SEM. *p<0.05, **p<0.01, ***p<0.005, ****p<0.001

**Figure S3 (A-B)** Scatter plot of clone formation assay of the rescue experiment in CC cells. **(C-D)** Transwell assays of the rescue experiment in CC cells. Scale bar = 50μm. The data was presented as means ± SEM. *p<0.05, **p<0.01, ***p<0.005, ****p<0.001

**Figure S4 (A)** GO enrichment of the DEGs from HeLa cell sequencing were performed. **(B)** Scatter plot of active RhoA and ROCK1 protein gray value analysis in CC cells after RacGAP1 knockdown or overexpressed. **(C)** Western blot analysis of MKK4, p-MKK4, MKK7, p-MKK7, Erk, p-Erk, p38 and p-p38 after RacGAP1 knockdown or overexpressed in CC cells. **(D)** Changes of p-c-Jun based on c-Jun after RacGAP1 knockdown or overexpressed in CC cells. **(E)** Corresponding gray value analysis in in CC cells after RacGAP1 knockdown or overexpressed. **(F)** Western blot analysis of JNK and p-JNK expression of three CC cell lines transfected with miR-192 inhibitor or mimics. **(G)** Changes of p-c-Jun based on c-Jun after transfected with miR-192 inhibitor or mimics in CC cells. **(H)** Corresponding gray value analysis in CC cells after transfected with miR-192 inhibitor or mimics. The data was presented as means ± SEM. *p<0.05, **p<0.01, ***p<0.005, ****p<0.001

**Figure S5** **(A)** Representative images of IHC staining of p-JNK, c-Jun, p-c-Jun, c-Myc in xenograft tumor tissues (Magnification, ×400). IHC scores of each group. **(B-C)** Corresponding gray value analysis of the rescue experiment in CC cells. **(D)** Clone formation assays of three CC cell lines treated with SP600125. **(E)** Relative expression of miR-192 of three CC cell lines treated with SP600125. **(F)** Corresponding gray value analysis in CC cells treated with SP600125. The data was presented as means ± SEM. *p<0.05, **p<0.01, ***p<0.005, ****p<0.001
